# The Wages of Imposition

**Published:** 1858-06

**Authors:** 


### THE WAGES OF IMPOSITION.

Two of the most splendid private mansions in New York, are owned and occupied by men who made their money by the sale of patent medicines. Philadelphia can say the same thing. Men of culture and wealth have their families attended by educated physicians of respectability and known worth, and so with the higher grades of the middle classes. It is upon the scanty earnings of the ignorant poor, that patent medicine harpies thrive; and but for their delusions, these poor creatures could have medical advice and medicine too, without pay. In the seventh Annual Report of the DEMILT Dispensary, corner of Second Avenue and Twenty-third Street, it is well said that, "One of the most miserable errors of the poorer classes is, the facility with which they are taken in by quack practitioners, and nostrums commonly called 'patent medicines.' The apparent advantages of cheap treatment, sometimes given by the rule of '*No cure no pay*,' and of preparations claimed to be newly invented or discovered, mysteriously compounded, extensively used, and always infallible, often prove too strong for these classes, and mislead them to their great injury, in impairing their constitutions, aggravating and prolonging their ailments, and little by little wasting their scanty means. Against that set of malign influences, a well-managed Dispensary, furnishing to all the poor within its territorial limits gratuitous assistance, both in the services of physicians, and in the supply of medicines, of the same character which the prosperous man provides for his own family, raises an effectual barrier. Cheap as quackery may be, the true medical art, applied without any charge, is still cheaper."

And yet there are multitudes of newspapers, secular and religious, who for a few dollars pay have published to the world, as "*the true* benefactors of mankind," the names of men who make their living by the sale of secret remedies for poverty's last mite; remedies which, if not utterly powerless of good, are inappropriate or absolutely destructive in their ultimate tendencies. Immeasurable, indeed, must be the gulf which separates these men and their money-bought adulators from the noble hearts whose surplus thousands and whose time are liberally devoted to the procurement of facilities by which the unfortunate children of poverty can have for the asking, the same medical counsel and remedial skill, which would, for the same ailments, be given to the wealthiest in the land; for among the attending physicians of the "DEMILT" are Doctors CAMMANN, PARKER, and DRAPER, who are second to none in New York, and who thus befriend the poor without fee or reward.

